# Unlocking high-performance near-infrared photodetection: polaron-assisted organic integer charge transfer hybrids

**DOI:** 10.1038/s41377-024-01695-9

**Published:** 2024-12-09

**Authors:** Muhammad Ahsan Iqbal, Xueqian Fang, Yasir Abbas, Xiaoliang Weng, Tingchao He, Yu-Jia Zeng

**Affiliations:** 1 School of Environment and Civil Engineering, Dongguan University of Technology, Dongguan, 523808 China; 2 Guangdong Provincial Key Laboratory of Intelligent Disaster Prevention and Emergency Technologies for Urban Lifeline Engineering, Dongguan University of Technology, Dongguan, 523808 China; 3 Department of Mechanics, Tianjin University, Tianjin, 300350 China; 4 Shenzhen Key Laboratory of Laser Engineering, Key Laboratory of Optoelectronic Devices and Systems of Ministry of Education and Guangdong Province, College of Physics and Optoelectronic Engineering, Shenzhen University, Shenzhen, 518060 China; 5 School of Mechanical Engineering, Dongguan University of Technology, Dongguan, 523808 China

**Keywords:** Optical sensors, Optoelectronic devices and components, Photonic devices

## Abstract

Room temperature femtowatt sensitivity remains a sought-after attribute, even among commercial inorganic infrared (IR) photodetectors (PDs). While organic IR PDs are poised to emerge as a pivotal sensor technology in the forthcoming Fourth-Generation Industrial Era, their performance lags behind that of their inorganic counterparts. This discrepancy primarily stems from poor external quantum efficiencies (*EQE*), driven by inadequate exciton dissociation (high exciton binding energy) within organic IR materials, exacerbated by pronounced non-radiative recombination at narrow bandgaps. Here, we unveil a high-performance organic Near-IR (NIR) PD via integer charge transfer between Poly[2,5-bis(3-tetradecylthiophen-2-yl)thieno[3,2-b]thiophene] (C-14PBTTT) donor (D) and Tetrafluorotetracyanoquinodimethane (TCNQF4) acceptor (A) molecules, showcasing strong low-energy subgap absorptions up to 2.5 µm. We observe that specifically, polaron excitation in these radical and neutral D-A blended molecules enables bound charges to exceed the Coulombic attraction to their counterions, leading to an elevated *EQE* (polaron absorption region) compared to Frenkel excitons. As a result, our devices achieve a high *EQE* of ∼10^7^%, femtowatt sensitivity (*NEP)* of ~0.12 fW Hz^-1/2^ along a response time of ~81 ms, at room temperature for a wavelength of 1.0 µm. Our innovative utilization of polarons highlights their potential as alternatives to Frenkel excitons in high-performance organic IR PDs.

## Abstract (web summary)

This groundbreaking study revolutionizes organic near-infrared photodetection by exploiting integer charge transfer mechanisms and harnessing polaron absorption, achieving unparalleled sensitivity (~0.12 fW Hz^-1/2^) and efficiency (~10^7^%) at 1.0 μm.

## Graphical abstract

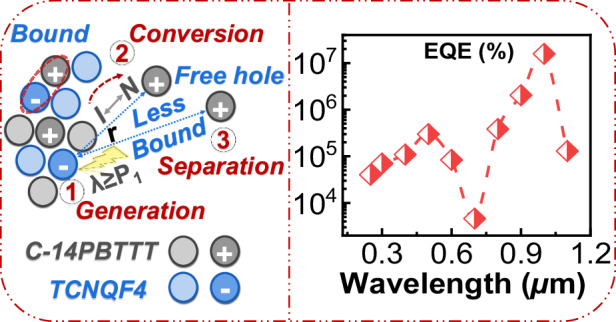

## Introduction

Infrared (IR) photodetectors (PDs), which are sensitive to wavelengths beyond 0.75 μm, are highly sought after for their applications in remote sensing, night vision, biological imaging, optical communication, etc^1^. Currently, commercial broadband (0.8–10 µm) IR PDs predominantly rely on inorganic semiconductors such as Ge, PbS, HgCdTe, and InGaAs, yet they encounter challenges related to cost, inflexibility, and intricate manufacturing processes^2^.

Organic photodetectors (OPDs), leveraging nanoscale organic thin films, are emerging as a prominent sensor technology set to revolutionize the Technological Revolution Era^3^. Organic semiconductors are promising for high-performance PDs owing to their strong light-matter interaction, mechanical flexibility, and seamless integration with silicon electronics through scalable solution processes^4^. However, recent endeavors to compete for the performance of commercialized inorganic PDs with IR OPDs have fallen short of achieving parity due to critical challenges^5^. The typical cut-off absorption wavelength of IR OPDs often falls below 1.0 µm, significantly limiting their use in broadband PDs^2^. Moreover, current state-of-the-art OPDs predominantly function within the ultraviolet-visible (UV–Vis) spectrum^6,7^, while existing IR OPDs exhibit notably low external quantum efficiencies (*EQE*)/responsivities (*R*), even under high external voltage^8–11^. This is attributed to inadequate exciton dissociation efficiency in organic IR materials, stemming from pronounced non-radiative recombination at narrow bandgaps^12,13^.

To address this challenge, researchers have employed the integration of IR-absorbing organic materials with high-mobility materials like graphene to enhance *R* via the photogating effect^14,15^. Despite achieving high *R* (~10^5^), the presence of a high density of trap states, attributed to the photogate effect, unfortunately, hampers response speed^16^. Furthermore, gapless graphene leads to substantial dark currents, posing a significant obstacle to achieving ultra-high IR sensitivity. Large dark currents in photodetection contribute to low sensitivity, strong low-frequency noise, and increased power consumption^17^. Consequently, the pressing need for innovative high-performance solitary organic materials in the IR spectrum has propelled the development of a new generation of IR OPDs. These advancements aim to achieve high *EQE* as well as ultra-high sensitivity in the IR spectrum, obviating the necessity for high-mobility layered hybrid structures^18,19^.

Among the two major types of charge transfer (CT) mechanisms between the donor (D) and acceptor (A) molecules, efficient low-energy absorption characteristics are often realized through integer charge transfer (ICT). In a *p*-type material, if the ionization energy (IE) of D < the electron affinity (EA) of A, D transfers an electron from its highest occupied molecular orbital (HOMO) to the lowest unoccupied molecular orbital (LUMO) of the A dopant, forming a donor cation (D^+^) and an acceptor anion (A^−^)^20,21^. The high degree of structural and energetic disorder induced by doping results in a broadening of the density of states (DOS)^22^. This structural distortion, along with the formation of the D^+^, defines a positive polaron. Moreover, the appearance of strong low-energy subgap (IR region) absorptions can be advantageous for the development of the next generation of IR OPDs.

However, by a widely accepted theory, the majority of formed polarons are coulombically bound by the A^−^^23,24^. In polymer/fullerene ICT blends, upon polaron excitation, the occurrence of charge-neutral excitations with weak interaction between spatially separated cation and anion radicals has been observed in polymers^25,26^. Similarly, polaron excitation in charged and neutral (1,1-bis(4-bis(4-methylphenyl)aminophenyl)cyclohexane) TAPC molecules enables bound holes to surpass the coulombic attraction to their MoO_3_ counterions, leading to an increased yield of free carriers^23^. Thus, an increase in photocurrent *EQE* may be achievable specifically through polaron excitation in blended radical ions of ICT-based D-A molecules^27^. This advancement could significantly impact IR OPDs, potentially leading to breakthroughs in the field.

Our study introduces a novel application of polaron excitations in a blend of radical and neutral ions within ICT-based D-A molecules to enhance NIR photodetection. We leverage polaron excitation to significantly boost NIR photodetection performance by employing a blend of C-14PBTTT polymer (D) and TCNQF4 (A) molecules to form a bulk type-II heterojunction within ICT-based systems. This approach exploits polaron absorption (~0.9–2.5 µm), which triggers a transition from bound to free polarons, surpassing traditional Frenkel exciton (F_ex_)-based mechanisms. The application of this strategy results in an *EQE* of ~1.57 × 10^7^% and a femtowatt sensitivity of ~0.12 fW Hz^−1/2^ at 1.0 µm, outperforming both recent NIR PDs and commercial IR PDs. Our work establishes a new experimental framework for leveraging polarons to enhance charge separation and transport in narrow bandgap materials, effectively reducing non-radiative recombination losses while maintaining the simplicity of device fabrication and structure. This demonstrates that significant performance improvements can be achieved using straightforward methods and commercially available materials, highlighting the potential of organic materials for advanced IR sensing and paving the way for future innovations in this field.

## Results

**Fig. 1:**
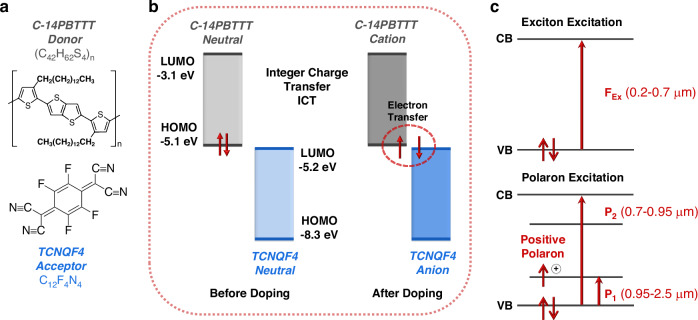
Mechanism of integer charge transfer (ICT) mediated doping between C14-PBTTT and TCNQF4. **a** Molecular structure. **b** Flat energy band diagrams before and after doping, **c** Distinction between Frenkel exciton (F_ex_) and polaron excitations (P_1_, P_2_)

**Fig. 2:**
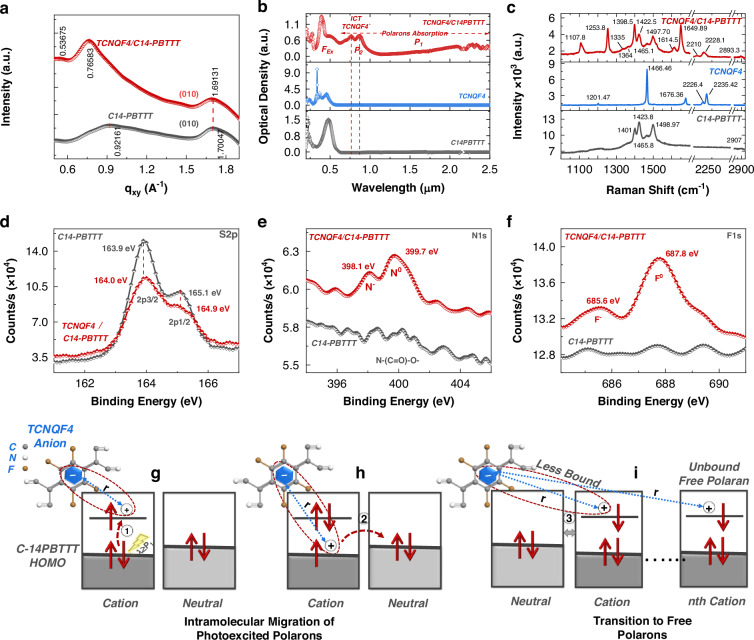
Doping spectroscopic analysis of C14-PBTTT, TCNQF4, and their TCNQF4/C14-PBTTT blend. **a** In-plane XRD pattern, **b** Uv-Vis-NIR spectra. **c** Raman spectra, **d**–**f** XPS spectrum, and **g**–**i** Intramolecular migration and transition to free polarons

The doping process involves the C-14PBTTT polymer acting as a D, while TCNQF4 dopant molecules function as A (Fig. 1a). In systems where the IE of a conjugated polymer D is lower than the EA of A, the doping mechanism follows the ICT model^20^, as illustrated in Fig. 1b. In our case, IE (C-14PBTTT) = 5.1 eV < EA (TCNQF4) = 5.2 eV. The HOMO and LUMO energy levels of C-14PBTTT and TCNQF4 molecules in solution, as depicted in Fig. 1b, are drawn based on well-established literature^21,28,29^. In this model, upon doping, an electron is transferred entirely from the D to the A, resulting in the creation of D^+^A^-^ on the polymer chain^30,31^. This localized structural distortion of the chain, coupled with D^+^ formation, defines a positive polaron^31,32^. This polaron formation is accompanied by the creation of a singly occupied energy level above the valence band edge and an unoccupied energy level below the conduction band edge^20,30^. These newly formed sub-gaps result in two strong subgap absorptions in the IR region, as illustrated in Fig. 1c (the corresponding labelled absorption peaks can be seen in Fig. 2b Uv-Vis-NIR spectra). These absorptions indicate the transition from the valence band edge to the half-filled subgap level (P_1_, 0.95–2.5 µm) and the transition from the lower to the higher subgap level (P_2_, 0.7–0.95 µm). In contrast, the transition from the valence band edge to the conduction band in the UV-Vis region (0.2–0.7 µm) is attributed to the transition of neutral molecules (F_ex_ transition). The pronounced vibronic progression observed in this region is a result of local exciton-vibrational coupling^31^.

The X-ray diffraction (XRD) analysis depicted in Fig. 2a provides valuable insights into the structural changes induced by doping in C14-PBTTT. The scattering peak associated with the π-stacking peak of C14-PBTTT, observed at *q*_xy_ = 1.70 Å^−1^, undergoes a shift following TCNQF4 doping. The downward movement of the in-plane (010) diffraction peaks suggests a contraction in the size of the π-stacking regions^28,33^. This contraction is likely driven by changes in the planarity of the polymer backbone, which can be attributed to the introduction of TCNQF4 dopants^34^. Furthermore, the data indicates that TCNQF4 molecules, used as dopants, predominantly localize in the alkyl side-chain regions of the C14-PBTTT polymer matrix. However, their presence significantly disrupts the π-π interaction between polymer chains^35^. This perturbation modifies the carrier concentration by introducing positive polarons into the material, thereby influencing its electronic and optoelectronic properties^21^.

In Uv-Vis-NIR absorption spectroscopy, optical transition characteristics can be indicative of ICT between D-A molecules^20^. The absorption spectra of C14-PBTTT, TCNQF4, and their blended solution, TCNQF4/C14-PBTTT, are depicted in Fig. 2b. Upon doping the C14-PBTTT polymer solution, its absorption peak centered ~0.48 μm, corresponding to a neutral absorption peak, diminishes, while subband gap transitions emerge ~0.82 μm. In the TCNQF4/C14-PBTTT spectra, peaks at 0.762 μm and 0.874 μm indicate the presence of the ionized TCNQF4 radical anions (localized charge) on top of the broadband positive polaron absorption (originally attributed to interchain polaron transitions from 0.95 to 2.5 μm)^21,33,36^. The distinct distinction between absorption features from mobile positive polarons (*p*-type doping) on the polymer (and anions) and undoped species (C14-PBTTT, TCNQF4) implies an ICT, rather than partial/hybrid CT^20,37^. Such a scenario would manifest as additional absorption features resulting from the formation of hybrid orbitals (new sub-bandgap absorptions) between C14-PBTTT and TCNQF4.

The verification of ICT doping between C14-PBTTT and TCNQF4 was substantiated through Raman spectroscopy. The alteration in the electronic structure of π-conjugated materials is closely linked with changes in the coordination of the π-core. Non-linear excitations, or polarons, significantly influence the vibrational properties. Consequently, Raman spectroscopy serves as a robust technique for probing doped polymers, offering valuable insights into their structural modifications and electronic interactions^38^. After doping, significant changes are observed in the neutral features. Specifically, Raman peaks at 1649.89 cm^−1^ and 2210 cm^−1^ correspond to the C=C stretching and C≡N stretching modes of the malononitrile group, respectively, indicating the anion state of TCNQF4. Raman peaks at 1335 cm^−1^ and 1364 cm^−1^ are associated with the cation state of C-14PBTTT^21,39,40^. Conversely, peaks at 1401 cm^−1^ and 1423.8 cm^−1^ correspond to the C=C stretching modes of the thienothiophene core and thiophene ring, respectively, indicating the neutral state of C-14PBTTT^21,38,39^. A detailed comparison is provided in the supplementary Information (Tables S1–3). In the TCNQF4/C14-PBTTT system, a finite fraction of neutral peaks remain, suggesting the coexistence of neutral and ionic molecules^39^. These changes in the Raman spectrum strongly support the occurrence of ICT upon TCNQF4 doping in C14-PBTTT.

X-ray photoelectron spectroscopy (XPS) was utilized to probe the core-level environment of atoms and ICT doping presence in TCNQF4/C14-PBTTT films, as illustrated in Fig. S1a. The spectrum reveals that the intensity of the C1s peak (284.8 eV)^41^ in TCNQF4/C14-PBTTT decreases compared to C14-PBTTT, while the binding energy (BE) of these peaks remains largely unchanged (Fig. S1b). This observation suggests that doping did not significantly alter the core-level environment of C1s atoms^7^. In the TCNQF4/C14-PBTTT spectrum (Fig. 2d), the peak intensity of the S 2p decreases compared to C14-PBTTT, while the BE of S 2p_3/2_ undergoes a blue shift of 0.1 eV (164 – 163.9 eV = 0.1 eV)^42,43^. One possible explanation is that the highly electronegative nitrogen induces a charge redistribution in the sulfur atoms, leading to an increase in BE indication of cation formation^44^. Furthermore, the appearance of the N 1 s peak observed in the TCNQF4/C14-PBTTT spectrum (Fig. 2e) was deconvoluted to reveal two distinct peaks: an ionized nitrogen peak centered at 398.1 eV (N^−^) and a neutral nitrogen peak at 399.7 eV (N^0^), which can be attributed to the malononitrile group. Similarly, the F 1 s peak (Fig. 2f) was deconvoluted into an ionized fluorine peak centered at 685.6 eV (F^−^) and a neutral fluorine peak at 687.8 eV (F^0^)^36,37,45^.

These spectroscopic analyses collectively indicate successful ICT doping, resulting in the formation of new low-energy (IR) CT bands between C14-PBTTT and TCNQF4. Furthermore, the analyses suggest the coexistence of both neutral and ionic molecule states coexist in the doped system. Based on these spectroscopic analyses presented, we propose a working mechanism for photo-induced CT in TCNQF4/C14-PBTTT blend under illumination. Traditional OPDs function by utilizing photoexcited F_Ex_, which disintegrate into electrons and holes at the D-A interface, subsequently collected by their respective electrodes. In contrast, the envisaged TCNQF4/C14-PBTTT blend device will operate on the principle of photoexcited polarons, initiating a photocurrent through two consecutive steps:

### Intramolecular migration of photoexcited polarons

The proposed mechanism stems from a recent investigation into photophysical phenomena, specifically focusing on photocurrent generation by polaron absorption in a doped small molecule^23^. Energy-level diagrams, as depicted in Fig. 2g–i, elucidate the initial stage of the photocurrent generation process. Within a doped polymer, each polymer chain is composed of both a neutral unit and a doped radical unit (because of low doping concentration). Upon exposure to IR light matching the energy level of the P_1_ transition, the electron located at the valence band edge of the radical unit undergoes excitation to the singly occupied subgap level, effectively raising the polaron to the valence band edge (Fig. 2g). Subsequently, hole transfer occurs from the radical unit to the adjacent neutral unit (Fig. 2h), driven by the energy differential between the valence band maximum and the external bias. This exchange transforms the neutral unit into a radical unit and vice versa, allowing polarons to migrate to neighboring neutral units when illuminated by the light of P_1_ energy^23,46,47^.

### Transition to free polarons

The subsequent stage involves a transition from a bound to a free polaron state, which is accountable for the generation of free charge carriers^23,48^. The Coulomb BE equation is represented as ${BE}\left(r\right)=\frac{{q}^{2}}{4\pi {\varepsilon }_{0}{\varepsilon }_{{\rm{r}}}r}$ where *q* denotes the elementary charge, *ε*_0_ signifies the permittivity of vacuum, *ε*_*r*_ stands for the dielectric constant of the host, and *r* represents the distance between the polaron and the corresponding anion radicals. If the bound polaron migrates multiple times until the *r* between the polaron and the stationary dopant anion exceeds BE under the influence of an external electric field, the polaron becomes a free hole, as depicted in Fig. 2i. Thus, the free hole can then be captured by the electrode, leading to the generation of enhanced photoconductivity. Consequently, as the total number and proportion of bound polarons increase, so does the sensitivity to IR light (P_1_)^23,36^.

**Fig. 3:**
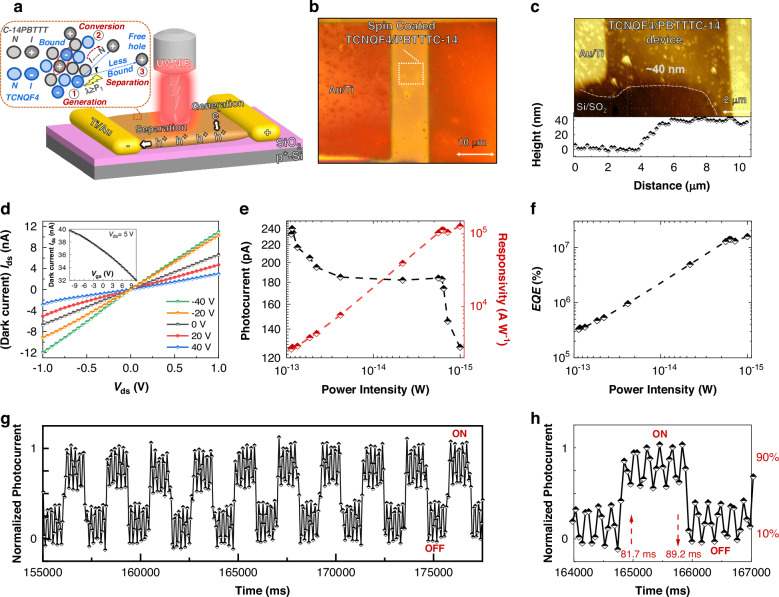
Microscopic and optoelectronic analysis of the TCNQF4/C14-PBTTT device at 1.0 μm. **a** Schematic of TCNQF4/C-14PBTTT phototransistor device, **b** optical image, **c** AFM image, **d** *I*_ds_ to *V*_gs_ curves in dark, **e** *I*_ds_ to *V*_ds_ curves in dark with different gate voltages (inset: *I*_ds_ to *V*_gs_ curves in dark), **e** *I*_ph_ dependence on light power intensities and corresponding *R*, **f** Light power intensities dependent *EQE*, **g** Photoresponse under light chopping, and (**h**) Photoresponse time measurement (85 fW) (*V*_g_ was 0 V and *V*_bais_ was 5 V)

Our phototransistor device was fabricated by spin-coating an optimized blend of TCNQF4 and C14-PBTTT onto a p^+^Si/SiO_2_ substrate (Fig. 3a), utilizing a bottom-gate/top-contact transistor architecture (fabrication process as depicted in Fig. S2). This ICT-based type-II bulk heterojunction comprises a solitary layer of radical and neutral TCNQF4/C14-PBTTT molecules, serving dual roles as the channel and the photoactive layer. As already discussed above, this process involves the excitation of electrons to the subgap level, followed by polarons (hole) transfer and subsequent liberation of bound polarons into free charge carriers under an external electric field (inset Fig. 3a), enhancing sensitivity to IR light. In Fig. 3b, an optical image of a TCNQF4/C-14PBTTT device with a 10 μm channel length is presented, showcasing uniformly spin-coated thin films. The corresponding AFM image, depicted in Fig. 3c, reveals the thickness of the TCNQF4/C-14PBTTT layer to be ~40 nm.

The electrical measurements depicted in Fig. 3d illustrate a *p*-type transport behavior observed during gate sweeping of the TCNQF4/C-14PBTTT device. The responsivity $R=\frac{I{\rm{ph}}\,}{P{\rm{d}}}$ of a PD is calculated as the ratio of photocurrent (*I*_ph_ *=* *I*_light, ON_ – *I*_light, OFF_) to the incident laser power per unit area (*P*_d_), which is a critical metric for PDs. $P{\rm{d}}=P{\rm{in}}{\boldsymbol{\times }}\frac{A{\rm{device}}\,}{A{\rm{spot}}}$ is derived from the total optical power (*P*_in_) and the ratio of the device area (*A*_device_) to the laser spot area (*A*_spot_). In Fig. 3e, the dependence of *I*_ph_ and *R* at 1.0 μm on light intensity is illustrated. At low-light levels (~1 fW), a peak *R* of ~1.27 × 10^5^ A W^−1^ is obtained, while at higher intensities (88.3 fW), a maximum *I*_ph_ of ~233 pA is recorded. The ${EQE}\left( \% \right)=\left(\frac{{hc}}{e\lambda }\right)\times R\times 100$ represents the ratio of circulating charge carriers to incident light photons. Here *c* represents the speed of light, *h* denotes the Planck constant, *e* signifies the electron charge, and *λ* indicates the incident laser wavelength. We have achieved a remarkable *EQE* of 1.57 × 10^7^% (Fig. 3f) with extremely low-light power of only 1 fW at 1.0 μm, indicating an exceptionally high yield in extracting photogenerated free carriers.

Response time is a critical parameter for assessing PD performance. It refers to the duration for the *I*_ph_ to transition from 10% to 90% (rise time, *τ*_r_) and from 90% to 10% (decay time, *τ*_f_) during the switching process under light exposure. The photoresponse of our device at 1.0 μm exhibits notable stability and repeatability across multiple cycles (Fig. 3g). At 1.0 μm illumination (Fig. 3h), we obtain *τ*_r_ of ~81.7 ms and *τ*_f_ of ~89.2 ms, demonstrating rapid response characteristics. We have been periodically measuring the devices over 6 months, which show good stability. Particularly, comparison tests on photocurrent switching curves were conducted over 11 weeks. The results consistently show high reproducibility across multiple cycles (Fig. S4), emphasizing the durability and reliability of our devices. The transit time (*τ*_transit_) of charge carriers can be calculated using ${\tau }_{{\rm{transit}}}=\frac{{L}^{2}}{\mu {V}_{{\rm{bias}}}}$ where *L* is the length of the transistor channel, *V*_bias_ is the source-drain voltage, and *µ* is the carrier mobility. For the TCNQF4/C-14PBTTT device, the calculated *τ*_transit_ is ~200 µs (Detailed calculations are available in the Supplementary Information).

**Fig. 4:**
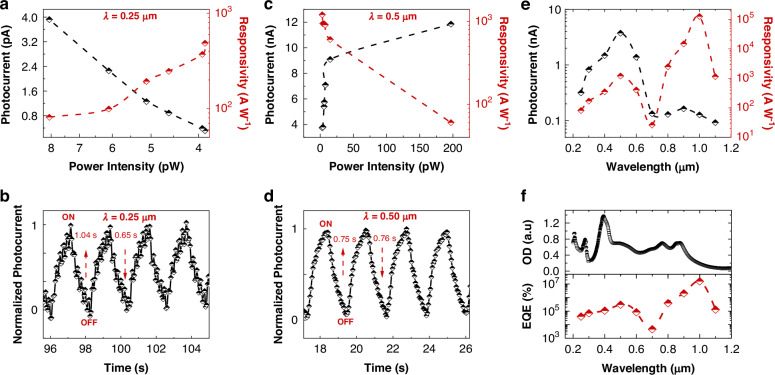
Optoelectronic measurements of the TCNQF4/C14-PBTTT device at other wavelengths. **a** Light power intensities dependent *I*_ph_/*R*, and **b** Photoresponse time measurement (4.11 pW) at 0.25 μm. **c** Light power intensities dependent *I*_ph_/*R*, and **d** Photoresponse time measurement (19 pW) at 0.5 μm. **e** Wavelength-dependent *I*_ph_/*R*_max_, and **f** Comparison of TCNQF4/C14-PBTTT blend absorption spectrum and *EQE* (*V*_g_ was 0 V and *V*_bais_ was 5 V)

In addition to the NIR wavelengths, we conducted detailed optoelectronic measurements of the TCNQF4/C14-PBTTT device at UV (0.25 μm) and Vis (0.5 μm) wavelengths. Under 0.25 μm light (Fig. 4a), we observe a peak *I*_ph_ of 3.92 nA at 8.07 pW and a maximum *R* (*R*_max_) of 81 A W^−1^ at 3.88 pW. Additionally, *τ*_r_ and *τ*_f_ show slower rates (Fig. 4b), ~1.04 s, and 0.658 s, respectively. Similarly, for 0.5 μm light illumination (Fig. 4c), we record a *R*_max_ of ~1.2 × 10^3^ A W^−1^ at a low-light intensity of 3.1 pW. Additionally, for 0.5 μm light, both *τ*_*r*_ and *τ*_*f*_ exhibit slower times, ~0.75 s and 0.767 s, respectively (Fig. 4d). Similarly, lower *EQE* at 0.25 μm and 0.5 μm wavelengths (Fig. S5b) is observed.

The wavelength-dependent lowest *I*_ph_ and their corresponding calculated *R*_max_ are presented in Fig. 4e at their respective minimum power intensities (Fig. S7) required for detection. It is noteworthy that the F_Ex_ (UV-Vis) wavelengths, exhibit a little higher *I*_ph_ but require corresponding higher power intensities for detection compared to P_1_(IR) wavelengths. Given the prolonged durations of *τ*_r_ and *τ*_f_ at F_Ex_ wavelengths, inherent photogating effects may be occurring. This phenomenon involves the trapping of photo-induced carriers at complex states and interfaces, thereby delaying carrier recombination and resulting in increased photogain^49–51^. On the other hand, P_1_(IR) wavelengths require minimal power intensities for detection, albeit with relatively lower *I*_ph_. This implies that a minimal number of photons can effectively trigger polaron excitation (P_1_), promote rapid intramolecular migration, and overcome Coulombic binding, finally resulting in ultrasensitive photoconduction.

The wavelength-dependent *EQE*, as depicted in Fig. 4f, showcases notable characteristics across different spectral ranges. The *EQE* ranges from 10^3^ to 10^5^% within the 0.25–0.7 μm range, with a peak of 10^7^% at 1.0 μm. The photoresponse shows a slight red shift, dipping ~0.7 μm, aligning with the absorption dip of the TCNQF4/C14-PBTTT blend at ~0.6 μm. The wavelength-dependent *EQE* closely matches the absorption spectrum of TCNQF4/C14-PBTTT, with visible range *EQE* attributed to F_Ex_ transitions within TCNQF4 or C14-PBTTT molecules. On the contrary, the peak *EQE* at 1.0 μm stems from the effective intramolecular migration of P_1_-excited polarons, transitioning into free polarons.

We have noted significantly faster response time (*τ*_r_ and *τ*_f_) characteristics at 1.0 μm (P_1_ region), suggesting distinct photo-induced charge carrier behaviors compared to those observed at ultraviolet (0.25 μm) and visible (0.5 μm) wavelengths (F_Ex_ region). To further investigate this we conducted a transient absorption (TAS) analysis of the TCNQF4/C14-PBTTT blend solution under visible and NIR regions. TAS provides information on the dynamics of photoexcited excitons and polarons, including their generation, transport, and recombination mechanisms.

**Fig. 5:**
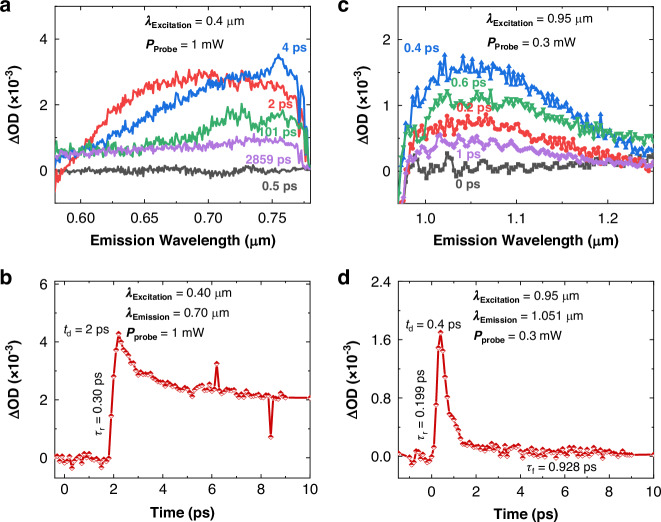
Transient and time-resolved absorption spectroscopic analysis (emission and excitation) of TCNQF4/C14-PBTTT blend. Under **a**, **b** Visible, and **c**, **d** NIR wavelengths

**Fig. 6:**
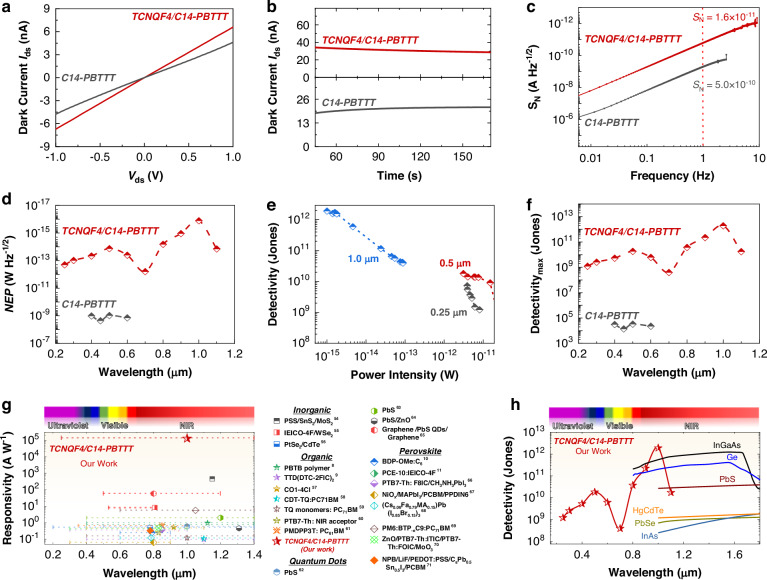
Specific detectivity (D*) measurements from dark currents of C14-PBTTT and TCNQF4/C14-PBTTT devices. **a** *I*_ds_ to *V*_ds_ curves, **b** *I*_ds_ to *t* curves, **c** Frequency-dependent noise spectral density (*S*_N_), **d** Wavelength-dependent *NEP*, **e** Light power intensities dependent D* at different wavelengths, **f** Wavelength-dependent peak D* (*V*_g_ = 0 V and *V*_bais_ = 5 V). Performance comparison of our detector wavelength-dependent: **g** *R* with recent NIR PDs (Such as Inorganic^54–56^, Organic^8,9,57–61^, Quantum dots^62–65^, Perovskite^10,11,66–71^ and featuring lines to denote their respective absorption ranges), and h) D* with commercial IR PDs (Such as PbS P9217-04, InAs P10090-01, PbSe P9696-03, InGaAs FGA21-CAL from Hamamatsu Photonics K. K., Japan^72^. Ge FDG03-CAL from Thorlabs, Inc., USA^73^

Transient and time-resolved absorption spectroscopic analysis of TCNQF4/C14-PBTTT blends reveal a peak emission (Fig. 5a) at 0.7 μm for 0.4 μm excitation (delay time = 2 ps). The charge separation time is 0.3 ps, and the separated excitons have a prolonged lifetime (Fig. 5b), confirming the presence of a photogating effect (consistent with photoresponse time measurements in Fig. 4d). Photogenerated charges trapped during the photogating effect do not effectively contribute to *I*_ph_, leading to energy losses and increased recombination rates^52^, where electron-hole pairs recombine and dissipate energy as heat. These factors degrade the signal-to-noise ratio, reducing sensitivity (detailed in Fig. 6) and making the photodetector unsuitable for high-speed detection^53^.

On the other hand, the peak emission at 1.051 μm for 0.95 μm excitation (Fig. 5c) shows an ultrafast charge separation time of 0.199 ps and a lifetime of 0.928 ps (Fig. 5d), indicating fewer trap states and efficient, rapid intramolecular migration of polarons. NIR photons can more efficiently generate charge carriers with less excess energy, reducing the likelihood of non-radiative recombination processes. Additionally, lower probe power intensity is required for NIR (0.3 mW) compared to visible wavelengths (1 mW), also indicating a better sensitivity.

The specific detectivity (D*) serves as a comprehensive merit for evaluating PDs, taking into account both *R* and dark noise. One prevalent form of noise is 1/*f* noise, stemming from fluctuations in carriers being trapped and released by defects and disorders. The spectral density of 1/*f* noise can be approximated using *S*(1/*f*) or *I*_N_ = |I(*f*)*|*^2^/(*F*_s_ *N*), where, I(*f*) signifies the discrete Fourier transform of the dark current waveform *I*(*t*), *N* represents the number of data points, and *F*_*s*_ denotes the sampling rate^15^. The *I*_ds_ to *V*_ds_ dark curve of the TCNQF4/C14-PBTTT device (Fig. 6a) exhibits an escalation in carrier concentration, as evidenced by the upsurge in dark current following TCNQF4 doping, suggesting an ICT doping mechanism. The comparison of sensitivity parameters between the C14-PBTTT and TCNQF4/C14-PBTTT devices is depicted in Fig. 6b–f.

Dark current and its corresponding current noise are shown in Figs. 6b and S5a. Noise spectral density (*S*_N_) is computed as *S*_N_ = √*I*_N_ (1/*f*). Typically, the *S*_N_ is assessed within a bandwidth of 1 Hz, which is commonly significantly lower than the detection bandwidth, facilitating direct comparison among PDs with varying bandwidth specifications. The TCNQF4/C14-PBTTT device exhibits a superior *S*_N_ value of ~1.64 × 10^−11^ A Hz^-1/2^ (Fig. 6c). The noise equivalent power (*NEP*) represents the ratio of *S*_N_ to *R* within a bandwidth of 1 Hz, as illustrated in Fig. 6d across various wavelengths. Our device has exhibited exceptional femtowatt sensitivity under NIR illumination, attributed to the efficient transition to free polarons, notably achieving ~0.12 fW Hz^−1/2^ at 1.0 μm.

D* characterizes the capacity of a PD to discern weak optical signals amid background noise. It is computed using the formula D^*^ = √*AB*/*NEP*, where *A* is the device area, and *B* is the electrical bandwidth of noise measurement. For the bare C14-PBTTT device, D* is ~10^5^ Jones in the visible region only. In contrast, for the TCNQF4/C14-PBTTT device, D* ranges from ~10^8^–10^12^ in the Uv-NIR spectrum. Its maximum D* is ~1.8 × 10^10^ Jones in the visible (0.5 μm) and ~1.9 ×10^12^ Jones in the NIR (1.0 μm). The significant difference in sensitivity parameters between the visible and NIR regions underscores the superiority of polaron excitons over Frenkel excitons for high-performance NIR photodetection.

## Discussion

Devices with faster response times often encounter a trade-off with reduced R values, and only a few achieve a competitive D* compared to our devices (see Table S5 for a detailed parameter comparison). In CMOS or CCD sensors, high responsivity is prioritized to maximize photon capture in low-light scenarios, optimizing image fidelity despite potentially compromised temporal resolution. This trade-off is permissible in applications such as long-exposure photography or astrophysical imaging, where enhanced photodetection precision is critical over rapid temporal response.

The performances of our TCNQF4/C14-PBTTT device are evaluated across different wavelengths, comparing its *R* with recent NIR PDs (Fig. 6g) and its D* with commercial IR PDs (Fig. 6h). Our device shows superior and well-balanced performance compared to recent studies and its D* is competitive with commercially available IR PDs. Overall, this polaron-assisted mechanism in organic ICT-based D-A molecules enhances NIR *EQE*/*R*, demonstrating the potential of organic materials to surpass traditional inorganic counterparts and advancing future innovations in IR sensing.

In conclusion, this study demonstrates groundbreaking femtowatt sensitivity by leveraging the ICT mechanism, particularly polaron excitations, in radical and neutral TCNQF4/C14-PBTTT blends. This high efficiency arises from the effective and rapid intramolecular migration of IR-excited polarons, transitioning into free polarons. The fabricated TCNQF4/C14-PBTTT NIR OPD device exhibits well-balanced, remarkable femtowatt optoelectronic performance, with *R* of ~1.27 × 10^5 ^A W^-1^, *EQE* of ~1.57 × 10^7^%, and D* of ~1.9 × 10^12^ Jones at 1.0 µm, outperforming traditional inorganic detectors. Moreover, the device demonstrates rapid response characteristics, with response times (*τ*_r_, *τ*_f_) of ~81.7 and ~89.2 ms, respectively. The notable divergence in performance parameters between the visible and NIR regions underscores the superior efficacy of polaron excitons relative to Frenkel excitons in facilitating high-performance NIR photodetection. These discoveries not only underscore the capacity of organic materials to outperform conventional inorganic alternatives but also lay the groundwork for future advancements in IR sensing technology.

## Materials and methods

### Materials

Poly[2,5-bis(3-tetradecylthiophen-2-yl)thieno[3,2-b]thiophene] (C14-PBTTT, 99%, PN#753971) and 2,3,4,5-Tetrafluorophenylacetonitrile (TCNQF4, 97%, PN# 376779) were purchased from Sigma-Aldrich (USA). Ortho-dichlorobenzene (o-DCB, >99.0%) was sourced from Tokyo Chemical Industry Co., Ltd. (Japan). Heavily *p*-doped Si/SiO_2_ substrates, with an oxide thickness of 300 nm, were provided by PrMat. (China). All other chemicals utilized in our experiments were of analytical grade and were employed without further purification.

### Preparation of TCNQF4/C14-PBTTT solution

TCNQF4 solutions at a concentration of 3 mg/mL were subjected to ultrasonic processing for 30 min after being dissolved in o-DCB and maintained at 50 °C. A C14-PBTTT polymer solution at a concentration of 5 mg/mL was prepared in o-DCB at 120 °C for 30 min and then held at 100 °C to prevent polymer gelation. Blending solutions were synthesized by adding a TCNQF4 solution (bright orange–red color) to the C14-PBTTT solution (bright red color), resulting in a color change to dark red or black depending on the weight percent (wt %) of TCNQF4 added. An optimized ratio of approximately C14-PBTTT ~ 4 repeats per TCNQF4 was achieved at 10 wt % (Molar Ratio of 0.25), causing the solution color to turn black. Subsequently, the blended solution was spin-coated for 60 s at 9999 rpm onto pre-cleaned Si/SiO_2_ substrates. The TCNQF4/C14-PBTTT mixed solutions were kept at 100–110 °C before spin casting to ensure the polymer and dopant remained in the solution and to achieve the desired thickness of the individual samples. The resulting films underwent annealing on a hotplate at 150 °C for 1 hour in a glovebox to remove residual solvent. A similar approach was employed to fabricate bare C14-PBTTT films without the addition of TCNQF4.

### Characterizations

XRD analysis was carried out using a D8 advanced X-ray diffractometer (Bruker, Germany). UV-Vis-NIR absorption spectra were obtained employing the Uv-Vis-NIR Spectrophotometer U-4100 (Hitachi, Japan). Molecular vibrational modes were observed using Raman spectroscopy with the Andor SR500 instrument (Andor Technology, England), operating a 532 nm excitation laser, while maintaining laser power below 0.6 mWm^−2^. Composition characterization utilized XPS with the Escalab 250xi instrument (Thermo Fisher, USA), featuring a monochromatic Al-Kα (1486.6 eV) source. Thickness measurements were performed in noncontact mode using an Atomic Force Microscope (AFM) on the SmartSPM 1000 (AIST-NT, USA). Transient absorption spectra were measured using a Newport transient absorption spectrometer (USA), equipped with a Spectra-Physics Solstice Ace regenerative amplifier (USA) (producing ~100 fs pulses at a 1.0 kHz repetition rate) for generating the probing beam supercontinuum and a TOPAS light converter for the pumping beam. Two different crystal sources were used to cover the visible (CaF_2_ crystal) and IR (YAG crystal) regions. During measurements, the 0.4 μm (or 0.95 μm) output pulses (1000 Hz, ~100 fs) from a Ti: sapphire regenerative amplifier were split into two parts. One part passed through a mechanical delay stage to pump a CaF_2_ crystal (or YAG crystal), generating a light continuum to serve as the probe pulses (~100 fs). The other part was directed into a TOPAS optical parametric amplifier to generate pump pulses (~100 fs) and was modulated at 500 Hz by an optical chopper. All samples were measured using air-free holders.

### Device fabrication

A copper TEM grid (GVSH, Gilder grid, UK) was utilized as a shadow mask to pattern TCNQF4/C14-PBTTT thin films on Si/SiO_2_ substrates before the deposition of metal electrodes. The metal electrodes, composed of Ti (10 nm) and Au (60 nm), were deposited using magnetron sputtering, resulting in channels with dimensions of 10 μm in length and 100 μm in width. Figure S2 illustrates the step-by-step procedure for device fabrication.

### Optoelectronic measurement system

All photocurrent measurements were conducted at standard room temperature in an ambient atmosphere. The diameter of the illuminated area was ~10 mm. The apparatus for photocurrent measurement consists of the following primary components: (1) A semiconductor characteristic analyzer (4200-SCS Keithley, Tektronix, Inc., USA); (2) A cage probe station; (3) Monochromator/Spectrograph (Omni-λ300i series, Zolix, China) equipped with dual gratings (1-240-250-600-NP and 1-120-400-1200-NP, Zolix, China) and light source comprising a 500-Watt Xenon lamp (GLORIA-X500A, Zolix, China); and (4) A power sensor (1919-R, MKS/Newport, Israel).

## Supplementary information

Supplementary Information

## Data Availability

The data supporting this study’s findings are available from the corresponding author upon reasonable request.
