# Supplementary material for: Unlocking high-performance near-infrared photodetection: polaron-assisted organic integer charge transfer hybrids
Source: Light Sci Appl. 2024 Dec 9;13:318. doi: 10.1038/s41377-024-01695-9 (PMC11625827; doi:10.1038/s41377-024-01695-9)
Supplement: Supplementary file 1 — Supplementary Information [file 41377_2024_1695_MOESM1_ESM.docx]

**Supplementary Information for**

**Unlocking High-Performance Near-Infrared Photodetection: Polaron-Assisted Organic Integer Charge Transfer Hybrids**

*Muhammad Ahsan Iqbal, Xueqian Fang ^*^, Yasir Abbas, Xiaoliang Weng,* *Tingchao He, and Yu-Jia Zeng^*^*

Dr. M. A. Iqbal, Prof. X. Fang

School of Environment and Civil Engineering, Dongguan University of Technology, Dongguan, 523808, China

Guangdong Provincial Key Laboratory of Intelligent Disaster Prevention and Emergency Technologies for Urban Lifeline Engineering, Dongguan University of Technology, Dongguan, 523808, China

Department of Mechanics, Tianjin University, Tianjin 300350, China

Y. Abbas

School of Mechanical Engineering, Dongguan University of Technology, Dongguan, 523808, China

Dr. M. A. Iqbal, X. Weng, Prof. T. He, Prof. Y.-J. Zeng

Key Laboratory of Optoelectronic Devices and Systems of Ministry of Education and Guangdong Province, College of Physics and Optoelectronic Engineering, Shenzhen University, Shenzhen, 518060, China

E-mail: studxfang@yeah.net; yjzeng@szu.edu.cn

*Corresponding author.

**Figure S1.** a-b) XPS spectra of C14-PBTTT and TCNQF4/C14-PBTTT films.

**Table S1**. Raman mode assignment for TCNQF4 states.^1, 2, 3^

| **Modes** | **Raman shift (cm^-1^)** | **Assigned Material** | **TCNQF4** **State** |
| --- | --- | --- | --- |
| **C-CN stretching** | 1466.4 | TCNQF4 | Neutral |
|  | 1465.1 | TCNQF4/C-14PBTTT |  |
|  | 1443 | --- | Anion |
| **C=C stretching** | 1676.36 | TCNQF4 | Neutral |
|  | 1649.89 | TCNQF4/C-14PBTTT | Anion |
| **C≡N stretching** | 2226.4 | TCNQF4 | Neutral |
|  | 2228.1 | TCNQF4/C-14PBTTT |  |
|  | 2210 | TCNQF4/C-14PBTTT | Anion |

**Table S2.** Raman mode assignment for neutral C-14PBTTT.^2, 3, 4^

| **Modes** | **Raman shift (cm^-1^)** | **State** |
| --- | --- | --- |
| **C=C stretching**  **thienothiophene core** | 1401 | Neutral |
| **C=C stretching**  **thiophene ring** | 1423.8 | Neutral |
| **C=C stretching/**  **C-C stretching/**  **shrinking** | 1498.97 | Neutral |

**Table S3.** Raman mode assignment for cation C-14PBTTT.^2, 3^

| **Raman shift (cm^-1^)** | **State** |
| --- | --- |
| 1335 | cation |
| 1364 | cation |

**Figure S2.** Fabrication process of TCNQF4/C14-PBTTT phototransistor device.

**Figure S3.** Comparison of light power intensities dependent *I*_p_ at 1.0 μm. (*V*_bais_ was 5 V). The light was chopped manually at irregular time intervals. Measurement was taken under slower frequency conditions.

**Figure S4.** Performance comparison of photoresponse under light (1.0 μm) chopping before and after 11 weeks. (85 fW) (*V*_g_ was 0 V and *V*_bais_ was 5 V).

**Figure S5.** a) *I*_ds_ to *V*_gs_ curves in the dark with different *I*_ds_, and b) Light power intensities dependent *EQE* at different wavelengths (*V*_g_ = 0 V and *V*_bais_ = 5 V).

**Figure S6.** a) Frequency-dependent current noise, and b) Light power intensities dependent *NEP* at different wavelengths (*V*_g_ = 0 V and *V*_bais_ = 5 V).

**Figure S7.** Minimum laser power intensity employed for their respective wavelengths.

***Mathematical calculations of photodetector parameters***

$$\mu=\frac{1}{Cg} X \frac{L}{W} X \frac{1}{V\mathrm{ds}} X \frac{dI\mathrm{ds}}{dVg}$$

……………………………(1)

Slope = d*I*_ds_/d*V*_g_ (TCNQF4/C14-PBTTT) = 370 pA V^-1^

Source to drain voltage = *V*_ds_ = 5 V

Length of channel = *L* = 10 µm = 1×10^-3^ cm

Width of channel = *W*=100 µm= 6.1×10^-3^ cm

*C*_g_ = 11.5 nF cm^−2^ is the capacitance per unit area of the 300 nm SiO_2_

Electron mobility (TCNQF4/C14-PBTTT) =*µ*= 1×10^-3^ cm^2^ V^-1^s^-1^

Transient time *= τ*_transit_ (TCNQF4/C14-PBTTT) = 200 µs

**Table S4**. Key photodetector performance parameters of the TCNQF4/C14-PBTTT device.

| **Wavelength** | **0.25 μm** | **0.50 μm** | **1.00 μm** |
| --- | --- | --- | --- |
| **Photocurrent**  **(*I*_p_)** | 315 pA-3.92 nA  @  3.88 pW-8.07 pW | 3.74 nA-11.85 nA  @  3.1pW- 197 pW | 127 pA-233pA  @  1 fW-88.3 fW |
| **Responsivity (*R*_max_)** | ~8.1×10^1^ A W^-1^  @  3.88 pW | ~1.2×10^3^ A W^-1^  @  3.1 pW | ~1.27×10^5^ A W^-1^  @  1 fW |
| **External quantum Efficiency**  **(*EQE*_max_ %)** | ~4 × 10^4^ | ~2.9 × 10^5^ | ~1.57 × 10^7^ |
| **Response**  **Time** | ***τ*_r_** = 1.04 s , ***τ*_f_** = 0.658 s | ***τ*_r_** = 0.75 s , ***τ*_f_** = 0.767 s | ***τ*_r_** = 81.7 ms,  ***τ*_f_** = 89.2 ms |
| **Noise Spectral Density**  **(*S*_N_)** | 1.64×10^-11^ A Hz^-1/2^  @  1 Hz | 1.64×10^-11^ A Hz^-1/2^  @  1 Hz | 1.64×10^-11^ A Hz^-1/2^  @  1 Hz |
| **Noise Equivalent Power**  **(*NEP*)** | 201 fW Hz^-1/2^ | 13.5 fW Hz^-1/2^ | 0.12 fW Hz^-1/2^ |
| **Specific Detectivity**  **(*****D**)** | 1.21 ×10^9^ Jones | 1.80 ×10^10^  Jones | 1.90 ×10^12^  Jones |

**Table S5**. Performance comparison of NIR-based photodetectors for different types.

| **Type** | **Device structure** | **Spectral Range**  **(nm)** | ***λ*_max_ (nm)**  **@**  **NIR** | ***R*_max_**  **(A W^-1^)** | ***D*_max_*/**  **(Jones)** | ***(τ*_r_*/τ*_f_*)***  ***sec*** | ***Year*** | ***Ref.*** |
| --- | --- | --- | --- | --- | --- | --- | --- | --- |
| **Inorganic** | PSS/SnS_2_/MoS_2_ | 1150 | 1150 | 4.57 × 10^2^ | 2.07 × 10^12^ | --- | 2023 | ^5^ |
|  | IEICO-4F/WSe_2_ | 532 - 808 | 808 | 8.32 × 10^0^ | 4.65 × 10^11^ | 3.39/4.24 m | 2022 | ^6^ |
|  | PtSe_2_/CdTe | 200 - 2000 | 780 | 5.06 × 10^-1^ | 4.20 × 10^11^ | 8.1 μ | 2018 | ^7^ |
| **Organic** | PBTB polymer | 400 - 2000 | 1122 | 9.60 × 10^-1^ | ~10^10^ | --- | 2020 | ^8^ |
|  | TTD(DTC-2FIC)_2_ | 400 - 1400 | 1100 | 9.50 × 10^-2^ | ~10^11^ | 42/434 μ | 2024 | ^9^ |
|  | CO1-4Cl | 400 - 1100 | 920 | 5.30 × 10^-1^ | 10^12^ | --- | 2020 | ^10^ |
|  | CDT-TQ: PC_71_BM | 600 - 1400 | 1100 | 1.10 × 10^-1^ | 2.40 × 10^10^ | --- | 2024 | ^11^ |
|  | TQ monomers: PC_71_BM | 400 - 1500 | 1000 | 1.20 × 10^-1^ | 2.00 × 10^11^ | --- | 2020 | ^12^ |
|  | PTB7-Th: NIR acceptor | 300 - 1000 | 830 | 2.50 × 10^-1^ | 2.20 × 10^12^ | 10.5 μ | 2020 | ^13^ |
|  | PMDPP3T: PC_61_BM | 350 - 1000 | 850 | 6.80 × 10^-1^ | 3.20 × 10^11^ | <150 μ | 2020 | ^14^ |
|  | Graphene/TCNQ | 600 - 2000 | 1000 | 2.00 × 10^3^ | --- | --- | 2018 | ^15^ |
|  | Graphene/P3HT-F4TCNQ | 600 - 1500 | 1000 | 6.00 × 10^5^ | --- | --- | 2019 | ^16^ |
|  | Graphene/TTF-CA | 500 - 2500 | 1000 | 5.00 × 10^5^ | ~10^11^ | --- | 2020 | ^17^ |
|  | ITO/ZnO/BTzIC/  TBzIC /MoO_3_/Ag | 350 - 1250 | 1100 | 3.70 × 10^-1^ | 2.24 × 10^13^ | 20/10 μs | 2024 | ^18^ |
|  | *TCNQF4/C14-PBTTT* | *250 - 2500* | *1000* | *1.27 × 10^5^* | *1.90 × 10^12^* | *81.7/89.2 m* | *2024* | ***This work*** |
| **Colloidal quantum dots** | PbS | 500 - 1400 | 1000 | 6.00 × 10^-1^ | ~10^12^ | --- | 2019 | ^19^ |
|  | PbS | 400 - 1600 | 1200 | 2.00 × 10^1^ | 4.00 × 10^12^ | 1/1.5 m | 2020 | ^20^ |
|  | PbS/ZnO | 400 - 1500 | 1310 | 4.60 × 10^-1^ | 4.10 × 10^11^ | 4.3/0.3 | 2020 | ^21^ |
|  | Graphene /PbS QDs/ Graphene | 500 - 1200 | 800 | 5.80 × 10^1^ | 2.00 *×* 10^11^ | --- | 2017 | ^22^ |
| **Perovskite** | BDP-OMe:C_60_ | 600 - 900 | 790 | 2.48 × 10^-1^ | 1.10 × 10^12^ | 11.0/5.6 μ | 2024 | ^23^ |
|  | PCE-10:IEICO-4F | 400 - 1000 | 850 | 2.50 × 10^-4^ | 1.10 × 10^12^ | 2.4/2.8 m | 2023 | ^24^ |
|  | PTB7-Th: F8IC/CH_3_NH_3_PbI_3_ | 300 - 1000 | 850 | 3.70 × 10^-1^ | 2.00 × 10^11^ | ---/5.6 n | 2020 | ^25^ |
|  | NiO_x_/MAPbI_3_/PCBM/PPDIN6 | 300 - 900 | 800 | 6.37 × 10^-2^ | 1.27 × 10^12^ | 12.7/6.9 μ | 2021 | ^26^ |
|  | (Cs_0.06_FA_0.79_MA_0.15_)Pb (I_0.85_Br_0.15_)_3_ | 500 - 800 | 785 | 1.20 × 10^-1^ | 1.50 × 10^12^ | 49/27 m | 2020 | ^27^ |
|  | PM6:BTP_-e_C9:PC_71_BM PhotoMulti | 600 - 1400 | 1050 | 5.60 × 10^0^ | 1.60 × 10^9^ | ---/1000 μ | 2020 | ^28^ |
|  | ZnO/PTB7-Th:ITIC/PTB7-Th:FOIC/MoO_3_ | 300 - 1000 | 830 | 4.80 × 10^-1^ | 2.58 × 10^11^ | 26/27 n | 2020 | ^29^ |
|  | NPB/LiF/PEDOT:PSS/CsPb_0.5_Sn_0.5_I_3_/PCBM | 700 - 950 | 850 | 2.70 × 10^-1^ | 5.42 × 10^14^ | --- | 2020 | ^30^ |

**References**

1. Le, T.H., et al. Identification of TCNQF4 redox levels using spectroscopic and electrochemical fingerprints (TCNQF4= 2, 3, 5, 6-tetrafluoro-7, 7, 8, 8-tetracyanoquinodimethane). *Inorganica Chim. Acta* **395**, 252-254 (2013).

2. Yamashita, Y., et al. Efficient molecular doping of polymeric semiconductors driven by anion exchange. *Nature* **572**, 634-638 (2019).

3. Fujimoto, R., et al. Molecular doping in organic semiconductors: fully solution-processed, vacuum-free doping with metal–organic complexes in an orthogonal solvent. *J. Mater. Chem. C* **5**, 12023-12030 (2017).

4. Francis, C., et al. Raman spectroscopy and microscopy of electrochemically and chemically doped high-mobility semiconducting polymers. *J. Mater. Chem. C* **5**, 6176-6184 (2017).

5. Dwivedi, A.K., et al. High-Responsivity PEDOT:PSS/SnS/MoS Double-Heterostructure-Based Organic– Inorganic Broadband Photodetector. *IEEE Trans. Electron Devices* **70**, 4694 - 4699 (2023).

6. Zhu, Q., et al. High performance IEICO-4F/WSe2 heterojunction photodetector based on photoluminescence quenching behavior. *Nano Res.* **15**, 8595-8602 (2022).

7. Wu, D., et al. Design of 2D layered PtSe2 heterojunction for the high-performance, room-temperature, broadband, infrared photodetector. *ACS Photonics* **5**, 3820-3827 (2018).

8. Lv, L., et al. Flexible short-wave infrared image sensors enabled by high-performance polymeric photodetectors. *Macromol.* **53**, 10636-10643 (2020).

9. Zhang, H., et al. Near-Infrared Organic Photodetectors with Spectral Response over 1200 nm Adopting a Thieno [3, 4-c] thiadiazole-Based Acceptor. *ACS Appl. Mater. Interfaces* **16**, 9088–9097 (2024).

10. Huang, J., et al. A high‐performance solution‐processed organic photodetector for near‐infrared sensing. *Adv. Mater.* **32**, 1906027 (2020).

11. Bills, T., et al. A General Strategy for Enhancing Sensitivity and Suppressing Noise in Infrared Organic Photodetectors Using Non‐Conjugated Polymer Additives. *Adv. Funct. Mater.*, 2314210 (2024).

12. Verstraeten, F., et al. Efficient and readily tuneable near-infrared photodetection up to 1500 nm enabled by thiadiazoloquinoxaline-based push–pull type conjugated polymers. *J. Mater. Chem. C* **8**, 10098-10103 (2020).

13. Zhou, Y., et al. Simple Tricyclic-Based A-π-D-π-A-Type Nonfullerene Acceptors for High-Efficiency Organic Solar Cells. *ACS Appl. Mater. Interfaces* **14**, 6039-6047 (2022).

14. Yokota, T., et al. A conformable imager for biometric authentication and vital sign measurement. *Nat. Electron.* **3**, 113-121 (2020).

15. Cui, M., et al. Graphene–organic two-dimensional charge-transfer complexes: Intermolecular electronic transitions and broadband near-infrared photoresponse. *J. Phys. Chem. C* **122**, 7551-7556 (2018).

16. Iqbal, M.A., et al. Organic charge transfer complexes on graphene with ultrahigh near infrared photogain. *Nanotechnology* **30**, 254003 (2019).

17. Iqbal, M.A., et al. Ultralow‐transition‐energy organic complex on graphene for high‐performance shortwave infrared photodetection. *Adv. Mater.* **32**, 2002628 (2020).

18. Yin, B., et al. Sensitive Organic Photodetectors With Spectral Response up to 1.3 µm Using a Quinoidal Molecular Semiconductor. *Adv. Mater.* **36**, 2310811 (2024).

19. YousefiAmin, A., et al. Fully printed infrared photodetectors from PbS nanocrystals with perovskite ligands. *ACS Nano* **13**, 2389-2397 (2019).

20. Zhou, W., et al. Solution-processed upconversion photodetectors based on quantum dots. *Nat. Electron.* **3**, 251-258 (2020).

21. Wang, X., et al. Amorphous ZnO/PbS quantum dots heterojunction for efficient responsivity broadband photodetectors. *ACS Appl. Mater. Interfaces* **12**, 8403-8410 (2020).

22. Nian, Q., et al. Graphene/PbS-quantum dots/graphene sandwich structures enabled by laser shock imprinting for high performance photodetectors. *ACS Appl. Mater. Interfaces* **9**, 44715-44723 (2017).

23. Wang, Y., et al. Semitransparent Near‐Infrared Organic Photodetectors: Flexible, Large‐Area, and Physical‐Vapor‐Deposited for Versatile Advanced Optical Applications. *Adv. Funct. Mater.*, 2313689 (2024).

24. Kamijo, T., et al. A touchless user interface based on a near-infrared-sensitive transparent optical imager. *Nat. Electron.* **6**, 451-461 (2023).

25. Li, C., et al. Ultrafast and broadband photodetectors based on a perovskite/organic bulk heterojunction for large-dynamic-range imaging. *Light Sci. Appl.* **9**, 31 (2020).

26. Wang, J., et al. Self‐driven perovskite narrowband photodetectors with tunable spectral responses. *Adv. Mater.* **33**, 2005557 (2021).

27. Wang, B., et al. Boosting perovskite photodetector performance in NIR using plasmonic bowtie nanoantenna arrays. *Small* **16**, 2001417 (2020).

28. Li, N., et al. Tuning the charge blocking layer to enhance photomultiplication in organic shortwave infrared photodetectors. *J. Mater. Chem. C* **8**, 15142-15149 (2020).

29. Liu, J., et al. Highly sensitive, broadband, fast response organic photodetectors based on semi-tandem structure. *Nanotechnology* **31**, 214001 (2020).

30. Cao, F., et al. Bionic detectors based on low‐bandgap inorganic perovskite for selective NIR‐I photon detection and imaging. *Adv. Mater.* **32**, 1905362 (2020).
